# Clinical Role of Epigenetics and Network Analysis in Eye Diseases: A Translational Science Review

**DOI:** 10.1155/2019/2424956

**Published:** 2019-12-23

**Authors:** Michele Lanza, Giuditta Benincasa, Dario Costa, Claudio Napoli

**Affiliations:** ^1^Multidisciplinary Department of Medical, Surgical and Dental Sciences, Campania University Luigi Vanvitelli, Naples, Italy; ^2^Clinical Department of Internal Medicine and Specialistics, Department of Advanced Clinical and Surgical Sciences, University of Campania “Luigi Vanvitelli”, 80138 Naples, Italy; ^3^U.O.C. Division of Immunohematology, Transfusion Medicine and Transplant Immunology, Department of Internal Medicine and Specialistics, University of Campania “Luigi Vanvitelli”, Naples, Italy; ^4^IRCCS SDN, Naples, Italy

## Abstract

Network medicine is a molecular-bioinformatic approach analyzing gene-gene interactions that can perturb the human interactome. This review focuses on epigenetic changes involved in several ocular diseases, such as DNA methylation, histone and nonhistone post-translational modifications, and noncoding RNA regulators. Although changes in aberrant DNA methylation play a major role in the pathogenesis of most ocular diseases, histone modifications are seldom investigated. Hypermethylation in *TGM-2* and hypomethylation in *MMP-2/CD24* promoter genes may play a crucial role in pterygium development; hypermethylation in regulatory regions of *GSTP1* and *OGG1* genes appear to be diagnostic biomarkers of cataract; hypomethylation of TGF-*β*1 promoter may trigger glaucoma onset; hypermethylation of the *LOXL1* gene might be associated with pseudoexfoliation syndrome. A large panel of upregulated micro-RNAs (miRNAs), including hsa-hsa-miR-494, hsa-let-7e, hsa-miR-513-1, hsa-miR-513-2, hsa-miR-518c, hsa-miR-129-1, hsa-miR-129-2, hsa-miR-198, hsa-miR-492, hsa-miR-498, hsa-miR-320, hsa-miR-503, and hsa-miR-373,^*∗*^ may have a putative role in the development of retinoblastoma. Hypermethylation of H3K4 and hypomethylation of H3K27 at the *TGFBIp* locus are putative pathogenic mechanisms involved in corneal dystrophies. Determining how, where, and when specific epigenetic changes trigger ocular diseases may provide useful clinical biomarkers for their prevention, diagnosis, and management, as well as innovative drug targets. PF-04523655, a 19-nucleotide methylated double-stranded siRNA targeting the *RTP80* gene, showed a dose-related improvement in best-corrected visual acuity (BCVA) in patients affected by diabetic macular edema. The observed results support a clinical network-based research program aimed to clarify the role of epigenetic regulators in the development of ocular diseases and personalized therapy.

## 1. Introduction

**Figure 1:**
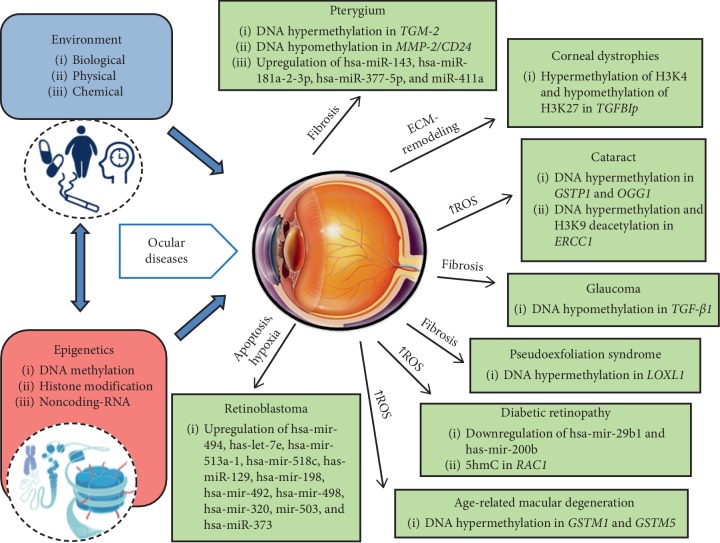
Epigenome: the bridge between environment and genome in ocular disease pathobiology. Environmental stimuli (exposome) interact with the genome through epigenetic modifications, which play a crucial role in controlling gene expression without changing the DNA sequence. The main epigenetic mechanisms are DNA methylation, histone modifications, and noncoding RNAs acting at different levels of the gene expression process. Aberrant epigenetic factors lead to common endophenotypes, including inflammation, fibrosis, proliferation, and adhesion that culminate in complex ocular diseases.

Epigenetics relies on modifications in gene expression induced by environmental alterations without changing the DNA structure [1]. The most common epigenetic changes include a crosstalk among DNA and mRNA methylation and histone and nonhistone modifications, as well as regulatory noncoding RNA molecules, mainly microRNAs (miRNAs), which may be meiotically (transgenerational effect) or mitotically inherited across several generations [1–4]. In contrast to genetic mutations, epimutations show a reversible nature. Small molecules, named epi-drugs, including the DNA methyltransferase inhibitors (DNMTi) and histone deacethyltransferase (HDAC) inhibitors, as HDACi, or activators, as HDACa, can revert aberrant epigenetic changes, thus suggesting the possible development of useful innovative drugs able to prevent or revert molecular defects underlying human diseases [5–7]. Improvements in next-generation sequencing (NGS) tools and bioinformatic algorithm development are providing the opportunity to trace subjects in a multilayered network by using “omics” data, in order to further dissect genome-environment interactions [8]. The ability to identify where, when, and how epigenetic sensors alter the homeostasis of specific cells or tissues may have a great clinical impact on patient management, from diagnosis and prognosis to customized therapeutic choices [8]. In ophthalmology, epigenetics is an emerging field, which is opening new routes to improve our knowledge of the molecular basis underlying physiological ocular development, as well as ocular disease onset [9, 10]. Disruption of several epigenetic-sensitive molecular networks may play a crucial role in the insurgence of the most frequent ocular diseases, such as pterygium, corneal dystrophies, cataract, glaucoma, diabetic retinopathy (DR), age-related macular degeneration (AMD), and retinoblastoma (Figure 1). Improving our knowledge of these complex processes might lead to the ambitious goal of identifying new noninvasive specific and sensitive predictors of risk and progression of ocular diseases, as well as new targets for future treatments [9, 10]. To date, no epigenetic-related biomarkers and therapies are currently used in clinical protocols and approved by the Food and Drug Administration (FDA), whereas several genetic tests for patients with inherited eye diseases, such as AMD, RB, and glaucoma, are exerting a profound influence over clinical practice and promise to transform personalized therapy [11]. This review focuses on the most current epigenetic-sensitive changes involved in the pathogenesis of ocular diseases in humans and discusses the impact of epigenetics in translating molecular networks into clinical settings. Furthermore, it suggests a network-based analysis as the most fruitful way to obtain useful applications in personalized therapy of ocular diseases. The most recent clinical investigations have been summarized focusing on the putative role of epigenetic-sensitive changes in modulating sensitivity and development of ocular diseases (see Table 1).

## 2. Literature Search Methods

Reviews, prospective randomized trials, prospective cohort studies, and retrospective studies of epigenetics in ophthalmology and the network medicine approach were searched using Medline through May 2019. Keywords in the search included epigenetics and eye disease, network medicine and ophthalmology, inflammation, oxidative stress, drug interactions, epigenetic regulators, DNA methylation/hydroxymethylation, histone/nonhistone post-translational modifications, and noncoding RNA regulators. Exclusion criteria included articles older than 20 years and those in languages other than English.

## 3. Ocular Surface and Corneal Diseases

### 3.1. Pterygium

Pterygium is a wing-shaped fibrovascular neoformation affecting the bulbar conjunctiva that may potentially extend over the corneal limbus. If it reaches the clear cornea, it may determine a reduction of visual acuity, mostly because of induced astigmatism [24]. The pathogenesis of pterygium is still unclear; however, among the possible causes, the following are recognized: stem cell deficiency, ultraviolet damage, metabolic disorders, neuronal alterations, and genetic dysfunctions [24]. Because of many differences in the methodology used in the papers published on pterygium, the prevalence of this disease appears to be very widespread. A recent meta-analysis shows that the overall prevalence of pterygium is 12% in the human population. Moreover, it is more frequent among older male subjects. Other demographic risk factors that appear to be important are outdoor occupations (mostly because of sunlight exposure) and rural residence [25]. An aberrant DNA methylation profile was detected in promoters of genes playing a pivotal role in pterygium pathogenesis [26]. By using human samples of pterygium, a locus-specific DNA methylation analysis of genes regulating matrix remodeling and cell adhesion was performed [26]. Evidence from this study reported significant hypermethylation in regulatory regions of the *transglutaminase 2* (*TGM-2*) gene, whereas sustained hypomethylation affected regulatory regions both in the *matrix metallopeptidase 2* (*MMP-2*) and the *CD24 molecule* (*CD24*) genes in patients with respect to the controls. This DNA methylation pattern was consistent with reduced levels of TGM-2, as well as increased levels of MMP-2 and CD24 proteins, contributing to fibroblastic and neovascular changes associated with pterygium formation [26]. Furthermore, some miRNAs, including hsa-miR-143-3p, hsa-miR-181a-2-3p, hsa-miR-377-5p, and hsa-miR-411a, resulted upregulated in primary pterygium fibroblasts compared with controls [12]. These results suggested that differential expression of these miRNAs is involved in the pathogenesis of pterygium; however, a larger number of patients are needed to ensure that the results obtained from the comparisons are sufficiently robust. Since pterygium treatment is mostly surgical with a high rate of recurrence, an adjuvant therapy targeting the aberrant methylation status of these three key genes might be extremely helpful in managing this disease.

### 3.2. Corneal Dystrophies

Granular corneal dystrophy type 2 (GCD2) is a rare inherited disease distinguished by superficial stromal deposits in the central cornea which can cause progressive visual drop [13]. GCD2 shows an autosomal dominant pattern of inheritance, for which heterozygous carriers do not affect central vision in a significant way, whereas the homozygous ones will determine a significant visual impairment from childhood [13]. GCD2-associated genetic alterations were mostly studied; as a result, the *transforming growth factor beta-induced* (*TGFBI*) gene is considered the master regulator of disease pathogenesis [27]. A study comparing wild-type (*n* = 3) vs heterozygous (*n* = 1) and homozygous (*n* = 3) GCD2 primary human corneal fibroblasts reported that epigenetic markers had a functional role in the TGF*β*1-mediated TGFBIp and extracellular matrix (ECM) gene expression, thus suggesting a key role in GCD2 onset [28]. In essence, hypermethylation of histone 3 at the level of lysine 4 (H3K4) as well as hypomethylation of histone 3 at the level of lysine 27 (H3K27) were significantly associated with activated regulatory regions in TGFBIp- and ECM-related loci in corneal fibroblasts isolated from patients compared with controls [28]. This evidence suggests that pharmacological manipulation of these histone modifications may be useful to obtain protective effects for granular corneal dystrophy. However, to date, no study has yet focused on this issue [28].

### 3.3. Keratoconus

Keratoconus (KC) is a corneal degeneration characterized both by progressive corneal prolapse and thinning, mostly affecting inferior and central sectors. Clinical evolution could lead to a decrease of vision because of high irregular astigmatism insurgence and/or development of corneal scars [14]. KC incidence ranges from 1/500 to 1/2000 subjects [14]. Although there is no particular connection with ethnicities or gender, this disease has been demonstrated to have an inheritance mechanism of transmission in 5–10% of cases, both autosomal recessive and dominant. Among the genes associated with KC, visual system home-box 1 (VSX1) has been demonstrated to be altered [29]. When we consider the minority of cases of KC actually connected with real gene inheritable alterations, it is easy to conceive this disease as a multifactorial one with many pathological mechanisms, both genetic and environmental, working in connection to determine the insurgence of KC [30]. Recently, the first KC-related transcriptome database, named KTCNlncDB (http://rhesus.amu.edu.pl/KTCNlncDB/), has been created [31]. Bioinformatic analysis of RNA-Seq has revealed several long noncoding RNAs strongly associated with disease etiology by affecting the expression of at least 996 genes in KC patients compared to healthy subjects [31]. In detail, the differentially regulated genes include cellular metabolism and fake regulator pathways, such as TGF-*β* and SMAD9, SMAD6, TGFB3, and TGFBR1 members of Hippo/Wnt signaling ways, which have already been associated with ocular health [31]. Understanding such modifications may open a novel window for therapeutic approaches in addition to current therapies. However, no clinical trials are in progress.

### 3.4. Cataract

Cataract is a progressive opacification of the crystalline lens of the eye which can determine a decrease in central vision and that is very common in the elderly [32]. It may be successfully treated by a surgical procedure that is very cost effective and successful, which restores the loss of vision and greatly improves the quality of life of the affected patients [33]. Accessing cataract surgery is not always easy, particularly for people living in rural and poor areas of developing countries. In such cases, cataract can be a permanent vision loss disease, becoming a major issue as an economic and social burden [34]. This is the reason why cataract is still the second leading cause of visual impairment and the first of blindness globally [34]. This disease represents 35% of all causes of blindness in the worldwide population, affecting 94 million people, 20 million of whom are blind because of cataract [34]. Among the causes of worldwide blindness around the world, age-related cataract (ARC) is certainly a major one [32–34]. ARC pathogenesis is still unclear, but it is considered a multifactorial one, with many elements involved in the disease onset, one of the most important ones being oxidative stress [32–34]. The lens is physiologically provided with molecular mechanisms to contrast oxidative stress and the related increase of reactive oxygen species in its structure, such as endogenous glutathione (GSH) activity and specific enzyme activity, with the aim of maintaining free thiols in the lens proteins [35, 36]. The action of GSH is helped by glutathione-S-transferases (GSTs), a group of proteins able both to catalyze the GSH action and to facilitate the transthiolation process [37].

*Glutathione S-transferase pi 1* (*GSTP1*) gene has been proven to be downregulated in ARC mainly because of epigenetic alterations [38]. In this study, in essence, the GSTP1 promoter was hypermethylated both in the lens epithelium and cortex from ARN patients compared with controls leading to strong reduction of GSTP1 levels; additionally, the grade of hypermethylation significantly correlated with severity of disease [38]. Furthermore, one of the most important repair actions from oxidative damage is performed by the *8-oxoguanine DNA glycosylase 1* (*OGG1*) gene both in the lens and in other human tissues [15]. A case-control study reported that CpG islands located in the first exon of the *OGG1* gene were hypermethylated in the lens cortex isolated from the ARC patients compared with controls, thus contributing to oxidative stress [15]. One of the most common causes of oxidative stress is ultraviolet radiation (UVR) because energy that is absorbed by the surface of the lens will damage the same tissue. However, the precise mechanism is still unclear [16]. One of the proteins playing a key role in DNA damage repair caused by UVR is Cockayne syndrome complementation group B (CSB), coded by the *excision repair 6 chromatin remodeling factor* (*ERCC6*) gene. A case-control study reported that in human UVB-treated LECs, the *ERCC6* gene was downregulated in ARC patients compared to controls, leading to an increased rate of apoptosis [39]. Essentially, both DNA hypermethylation and hypoacetylation of histone 3 lysine 9 (H3K9) at the promoter level contributed to increase the damage induced by UVB radiation [39]. This evidence suggests that by avoiding most of the causes of oxidative stress that usually damage the lens, it is possible to reduce the incidence of ARC caused by the epigenetic factors listed above.

## 4. Glaucoma and Pseudoexfoliation Syndrome

It is estimated that nearly 60 million people around the world are affected by the optic neuropathy known as glaucoma [17]. In this disease, there is progressive damage and death of retinal ganglion cell (RGC) axons, caused mostly by an increase of intraocular pressure (IOP); other factors involved are a decrease in ocular perfusion pressure, genetic alterations, and age [40]. Fibrosis is a well-known factor in the pathogenesis of glaucoma, resulting in increased ECM deposits both in the trabecular meshwork (TM) and in the lamina cribrosa (LC) [41]. Increasing the fibrosis in these two anatomical sites may lead to an increase in IOP because of clogging of TM and to damage directly the RGC, compressing nerve fibers by an enlarged LC. Together with fibrosis, hypoxia has also been demonstrated to have a crucial role in glaucoma development and its course [42]. The regulation of both fibrosis and of hypoxia processes would appear to be very important in glaucoma management. The cytokine transforming growth factor *β* (TGF *β*) family are involved in several cell mechanisms, among which is ECM production and accumulation in TM and LC [18, 43]. A study comparing the expression profile of cultured human LC cells reported that DNA hypomethylation in the promotor of the *TGF-β1* gene was responsible for its increased transcription in glaucomatous eye donors compared to controls [43]. This evidence suggests a crucial role for DNA methylation in glaucoma onset as well as in regulating *TGFβ1* gene expression as a novel therapeutic approach [43].

Pseudoexfoliation (PEX) syndrome is a disease closely connected to glaucoma because it is characterized by an overproduction and accumulation of fibrotic material in the TM [44, 45]. PEX glaucoma is harder to manage because usually IOP is higher and the course has a worse prognosis [46–48]. Although PEX is considered a multifactorial disease, two proteins have been detected as the major ones involved in its pathogenesis: *lysyl oxidase like 1* (*LOXL1*) and apolipoprotein E (APOE) [49]. Interestingly, a case-control study reported that the promoter region of the *LOXL1* gene was hypermethylated in patients with PEX compared with controls, leading to a reduced expression of its protein product and downstream impaired elastic fiber homeostasis [19]. However, studies are a long way from proposing a therapeutic strategy relying on epigenetic alterations in PEX [50].

## 5. Retinal Diseases

### 5.1. Diabetic Retinopathy

DR is one of the most frequent diabetes-related microvascular complications that can lead to irreversible blindness, if untreated [20, 51]. Following recent reviews [9], several experimental studies reported that hyperglycemia-induced epigenetic changes play a crucial role in developing DR. Recently, lower plasma levels of hsa-mir-29b1 and has-mir-200b have been associated with an increased risk of DR onset in type 2 diabetes (T2D) patients compared to controls [20]. This evidence points to these molecules as useful prognostic biomarkers or innovative drug targets. Other current experimental evidence arises from human retinal endothelial cells exposed to high glucose treatment [21]. In detail, the ten-eleven translocation (TET) enzyme caused increased levels of 5-hydroxymethyl cytosine (5hmC) on the *rac family small GTPase 1* (*RAC1*) gene promoter. This epigenetic modification led to an increased binding of the nuclear factor-kappa light beta chain (NF-kB), as a transcription factor able to activate RAC1 gene expression. As a downstream effect, ROS levels were largely increased, thus suggesting a crucial role for DNA hydroxymethylation in the development of DR [21].

### 5.2. Age-Related Macular Degeneration

Age-related macular degeneration (AMD) is a degenerative disease affecting the macula and many factors, both genetic and environmental ones, which are involved in its onset [52]. It is the most important disease causing an irreversible loss of visual acuity [53]. One of the peculiar alterations of AMD is the presence of deposits, made up of lipids and proteins, localized in the central retina that are known as drusen [54]. Moreover, it is possible to observe a constant degeneration of both retinal pigmented epithelium and neural retinal layer. Advanced stages of AMD can evolve into a large atrophic macular area, dry geographic atrophy (GA), or into a wet neovascularization of the macular area [54, 55]. Some epigenetic alterations have been observed in the insurgence of this disease [56]. A case-control study reported that the *glutathione S-transferase isoform mu1* (*GSTM1*) and *mu5* (*GSTM5*) genes were hypermethylated at the promoter level in AMD patients compared to controls [22]. This evidence suggests that GSTM1 and GSTM5 undergo epigenetic-sensitive repression, thus increasing susceptibility to oxidative stress in AMD retinas [22]. Furthermore, a genome-wide DNA methylation profile performed on peripheral blood cells showed a significant correlation between CpG methylation status and a single nucleotide polymorphism (SNP) harbored in the age-related maculopathy susceptibility 2 (ARMS2) gene in ADM patients compared to controls [57]. This evidence suggests a possible interaction between genetic and epigenetic changes, which synergistically trigger the disease development [57].

### 5.3. Retinoblastoma

RB is a rare kind of tumor, with an evaluated incidence range from 1 in 15000 to 18000 in new-born children, arising in the retinal cells [58]. Usually, this cancer affects only one eye but sometimes, depending on heritable factors, it may involve both eyes [59]. Alteration of cell division is the key factor for RB development, and a mutation of the retinoblastoma suppressor (RB1) gene has been demonstrated to be involved in this process [59]. Several studies have identified a role for RB1 in regulating many different epigenetic processes, such as DNA methylation and histone modifications [59–61]. Hypermethylation is one of the most common epigenetic alterations involved in this disease, thus affecting many genes, such as the *Von Hippel–Lindau tumor suppressor* (*VHL*) [62], the *cyclin-dependent kinase inhibitor 2A* (*CDKN2A/p16*) [63], and the *O6-methylguanine-DNA methyltransferase* (*MGMT*) genes [64]. Acetylation, phosphorylation, and methylation represent some of the most diffuse and important alterations that can occur in tumor onset such as RB [65]. In addition, miRNAs have been demonstrated to play a major role in cancer development [66, 67]. Alterations in a large panel of miRNAs, including highly expressed hsa-miR-494, hsa-let-7e, hsa-miR-513a-1, hsa-miR-513a-2, hsa-miR-518c, hsa-miR-129-1, hsa-miR-129-2, hsa-miR-198, hsa-miR-492, hsa-miR-498, hsa-miR-320, hsa-miR-503, and hsa-miR-373, may lead to expression of aberrations of connected genes, thus influencing RB development [23]. These alterations can promote tumor insurgence through increasing levels of hypoxia [68] inhibiting physiologic immunosuppression and reducing apoptosis of tumor cells [69, 70] as well as post-translational changes of nucleosomal histones [71, 72]. They can determine activation or inactivation of chromatin, assuming an important role in RB onset [71, 72]. Post-translational alterations of the RB protein (pRB) have been connected with many alterations in RB, even when the *RB1* gene is unaltered [73–75].

## 6. Small RNA-Based Therapeutics in Clinical Trials

RNA interference (RNAi) is a multistep process generating small interfering RNA, which mediates the degradation of its complementary RNA. Generally, RNAi is used for biomedical research, drug discovery, and treatment for many human diseases [76]. The eye is a confined compartment and enables the local delivery of siRNAs by topical instillation or direct injection, suggesting an optimal target for RNAi therapy [77]. In detail, PF-04523655, a 19-nucleotide methylated double-stranded siRNA is in phase 2 clinical trial (NCT00701181) for the treatment of diabetic macular edema [77]. From the results, PF-04523655 demonstrated a dose-related improvement in best-corrected visual acuity (BCVA) in patients with respect to controls, suggesting that further studies are needed to determine the optimal efficacious dose [66].

## 7. Network Medicine: A New Challenge for Personalized Therapy in Ophthalmology

### 7.1. Precision Medicine in Ocular Diseases

The most serious eye diseases have been proven to have a very complex nature: there are both a number of genetic factors and environmental influences leading to their insurgence and to their clinical characteristics [67]. This explains why the suffix “omics” appears more and more frequently in studies that try to unveil the insurgence mechanisms of eye diseases. It refers to a new kind of approach to molecular analysis of the biological components involved in mechanisms affecting the eye. Thanks to this approach, new biomarkers for early diagnosis may be identified and new targets to refine and to customize therapies might be recognized.

Genetics alone is not able to provide every answer to the complexity of eye diseases, and because of this, it requires integration with other new approaches such as transcriptomics, proteomics, metabolomics, and epigenomics. Genome-wide association studies (GWAS) have facilitated the majority of discoveries in genomics thanks to new techniques such as whole genome sequencing (WGS), microarrays targeting rare variants (exome chips), and whole exome sequencing (WES) [23, 68]. Transcriptomics is the study of the coding and noncoding RNA molecules codified by the whole genome, known as the transcriptome, by using RNA microarray analysis and RNA sequencing [69, 70]. Improvements in technology related to mass spectrometry devices have permitted more accurate data analysis of the overall biological proteins and molecules produced by a human organism. Thus, proteomics and metabolomics are emerging in recent years [71]. These new approaches have been mostly applied to AMD, glaucoma, and DR. In particular, many mechanisms of AMD insurgence have been recognized, thanks to GWAS [72]. The majority of eye diseases have multifactorial causes; this is the reason why the sectorial research of the precise mechanism of insurgence (genetic or environmental) has led to poor results. To merge the information which comes from the in-depth analysis permitted by genomics, proteomics, transcriptomics, epigenomics, and metabolomics will lead to a better understanding of every aspect involved in the etiology of each kind of disease [73]. The most important difficulty in this approach is to adopt the advanced kind of analysis required; this is not possible to perform in every research centre because an improvement of knowledge and instrumentation is required [74, 78]. On the contrary, it is important to recognize that new information may lead to greater improvements in the quality of life of most patients affected by eye diseases and to financial savings for national health systems.

### 7.2. Longitudinal Analyses in Ophthalmologic Clinical Research Programs

One of the most important challenges in advancing our knowledge of complex human diseases is to overcome the limitations related to nature and size of the experimental sample, the amount of information on environmental exposure and clinical phenotypes, and the difficulty in applying the basic findings to clinical follow-up, which are essential to obtain potential implications for diagnosis, prevention, and therapeutic interventions. The current omics tools allow us to affordably dissect the individual genetic background with a single nucleotide polymorphism (SNP) array or more expensively by using WGS. However, the extent/measurement of the magnitude of environmental exposure and epigenetic changes over time is more difficult to understand but it is imperative to determine in order to advance our knowledge of complex ocular diseases [67, 78]. A comprehensive staging approach should monitor individual variations from the fetal-perinatal stage to childhood, older age, and over several generations, thus providing earlier identification and management of at-risk subjects before the development of irreversible complex diseases [8]. Furthermore, the establishment of national initiatives known as “biobanks” recruiting up to millions of individuals permits the creation of a very rich database to measure multiple disease-associated phenotypes and objective symptoms in a consistent manner. Examples are the UK Biobank and the All of Us project in the USA accelerating discoveries of candidate genes, epigenetic and genetic mutations, and biological pathways, in order to build the path from genome to phenomena at an individual level. To date, the UK Biobank is one of the largest prospective cohorts worldwide with extensive open-access data on ophthalmic diseases and conditions [79]. Recently, unbiased empirical data from patients accruing in the UK Biobank cohort have indicated that these nested case-control studies have a strong statistical power in predicting potential genetic, lifestyle, and environmental determinants of AMP, cataract, and glaucoma [80]. Importantly, genetics and data from the UK Biobank are used by clinicians and academics forming the UK Biobank Eye and Vision Consortium (http://www.ukbiobankeyeconsortium.org.uk) aimed both to study molecular causes underlying eye diseases and vision loss and investigate how these can be controlled, prevented, and treated. The European Retinal Disease Consortium (ERDC) consists of 22 research groups, mostly from Europe, three from Israel, one from Canada, and one from the USA, which are focused on discovering inherited retinal disease- (IRD-) associated candidate genes. A list of rare sequence variants derived from WES data, clinical subtype, and the putative inheritance pattern is available on the website (https://www.erdc.info/index.php). Furthermore, the genetic and clinical data of IRD patients have resulted in many joint papers and several opportunities for collaboration. Interestingly, the EYE-RISK consortium involving 14 partners across Europe (http://www.eyerisk.eu/) aims at identifying risk factors, molecular pathways, and therapeutic approaches for the insurgence of AMD by using a systems medicine approach. The main goals are (1) development of robust algorithms utilizing genetic and nongenetic risk factors to identify personalized risks of AMD onset; (2) identification of novel biomarkers for further stratification of disease risks; (3) elaboration of preventive medical recommendations for high-risk subgroups of AMD patients; and (4) identification of molecular drivers/biological pathways relevant for onset and progression of advanced AMD that may be used to identify and validate new therapeutic targets. The European Eye Epidemiology (E3) consortium (http://eye-epi.eu) involves 31 groups originating from 13 different countries, which comprises both population-based, case-control and randomized trials providing ophthalmological data on about 170,000 European participants. The main goal is to identify risk biomarkers and molecular pathways underlying eye diseases, such as lifestyle, vascular and metabolic factors, genetics, and epigenetics, in order to develop and validate prediction models [81, 82]. Currently, the rate at which pathogenic omics data are being adopted in clinical practice is slow; however, large consortium efforts have the potential to improve the quality of clinical research and facilitate the translation of big data from bench to bedside.

### 7.3. Putative Network-Based Analysis to Identify Protein-Protein Interactions (PPIs) in Ocular Diseases

**Figure 2:**
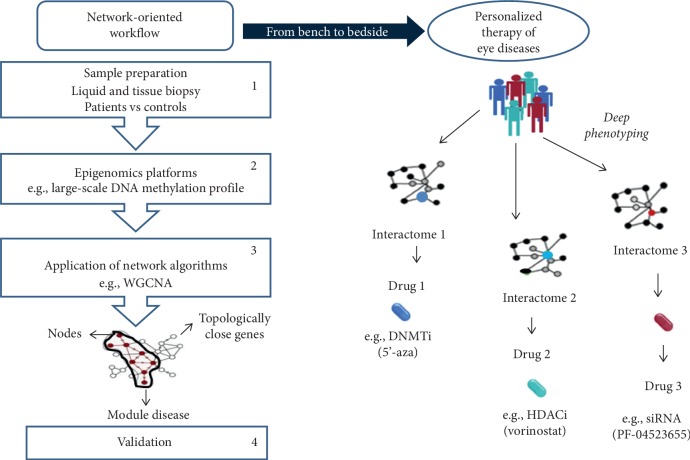
A network-oriented workflow. The computational pipeline relies on four steps: (1) sample preparation (patients vs controls), (2) whole-genome bisulfite sequencing, weighted coexpression network analysis (WGCNA), (3) constriction of network-based maps, and (4) validation and predictions. These epigenetic biomarkers and correlated interactomes may divide DR patients into precise groups, thereby improving personalized therapy.

This study discusses the putative contribution of the network medicine approach to advance personalized therapy of ocular diseases. Network medicine relies on potent bioinformatic tools analyzing multilayered disease phenotypes within the framework of molecular interaction (interactome), thus surpassing the current reductionist approach [6–8, 82]. The main goal is to discover novel molecular alterations able to alter the interactome flow and define the causative relationship between candidate genes and diseases [6–8, 82]. Remarkably, network medicine might redefine complex diseases by focusing on their underlying pathobiological molecules, leading to a new classification system in relation to the current reductionist approach. In this computational era, the analysis of network topological properties is performed by protein-protein interactions (PPIs), regulatory, and coexpression networks, which can be viewed as maps where disorders are represented with localized perturbation within a specific module (pathway) of the interactome [8]. The epigenetic fingerprint plays a key role in the onset of ocular diseases, and its reversible nature may have a great impact on therapeutic management. In Figure 2, we illustrate a useful network-oriented pipeline clinical research program aimed to analyze the large-scale DNA methylation profile in patients affected by eye diseases versus controls. The workflow shows four steps to discover epigenetic-related biomarker and putative drug targets: (1) sample preparation (e.g., peripheral mononuclear blood cells), (2) next-generation platforms, such as whole-genome bisulfite sequencing, (3) bioinformatic analysis with a network-based algorithm, such as the weighted coexpression network analysis (WGCNA), and (4) validation [8, 82]. By using deep molecular phenotyping, the most promising application of network medicine is personalized therapy. Epigenomic data, combined with clinical features, may help clinicians in identifying which eye of the patients will benefit from a given therapeutic strategy.

## 8. Conclusions

Epigenetics, and mainly DNA methylation, plays an important role in the pathophysiology of numerous ocular diseases. Owing to its reversible nature, epigenetic regulation could open a novel avenue for more effective therapeutic approaches, such as PF-04523655. However, clinical research is still in its beginning. A deeper understanding of the epigenetic fingerprint may enlarge opportunities to identify novel molecular biomarkers providing a more accurate stratification of at-risk population and customized therapies. Network medicine may assist in prioritizing novel putative candidate genes and designing innovative drug targets, and it represents the most fruitful way to reach precision medicine and personalized therapy for ocular diseases.

## Figures and Tables

**Table 1:** Pathogenic epigenetic modifications in different ocular diseases.

Disease	Epigenetic modification	Gene	Effect	Phenotypic outcome	References
Pterygium	DNA hypermethylation	*TGM2*	Reduced protein expression		
	DNA hypomethylation	*MMP2*	Increased protein expression	Fibrosis and neovascular changes	[12]
	DNA hypomethylation	*CD24*	Increased protein expression		
	Upregulation of hsa-miR-143a-3p, hsa-miR-181a-2-3p, hsa-miR-377-5p, and hsa-miR-411a	—	Reduced mRNA and protein expression	—	[13]

GCD2	H3K4 hypermethylation H3K27 hypomethylation	*TGFBIp*	Increased gene expression	Increased expression of ECM genes	[14]

Cataract	DNA hypermethylation	*GSTP1*	Reduced mRNA and protein expression	Increased oxidative stress	[15]
	DNA hypermethylation	*OGG1*	Reduced mRNA and protein expression	Increased oxidative stress	[16]
	DNA hypermethylation H3K9 hypermethylation	*ERCC6*	Reduced mRNA and protein expression	Increased rate of apoptosis	[17]

Glaucoma	DNA hypomethylation	*TGF-β1*	Increased protein expression	Increased ECM protein production and accumulation	[18]

PEX	DNA hypermethylation	*LOXL1*	Reduced protein expression	Failure of elastic fiber homeostasis	[19]

Diabetic retinopathy	Downregulation of hsa-miR-29b-1 and hsa-miR-200b	//	Overexpression of gene target	Deregulation of cellular survival/apoptosis, ECM cytoskeleton signaling	[20]
	5hmC	*RAC1*	Increased binding of NF-kB	Increased ROS levels	[21]

AMD	DNA hypermethylation	*GSTM1/5*	Reduced protein expression	Increased susceptibility to oxidative stress	[22]

RB	Upregulation of hsa-miR-494, hsa-let-7e, hsa-miR-513a-1, hsa-miR-513a-2, hsa-miR-518c, hsa-miR-129-1, hsa-miR-129-1, hsa-miR-129-2, hsa-miR-198, hsa-miR-492, hsa-miR-498, hsa-miR-320, hsa-miR-503, and hsa-miR-373^*∗*^	—	Deregulation of gene targets	Associated to insurgence/progression of retinoblastoma tumorigenesis through hypoxia, immune escape, reduction of apoptosis	[23]

TGM2: transglutaminase 2; MMP2: matrix metalloproteinase 2; CD24: CD24 molecule; TGFBIp: transforming grow factor B-induced; GSTP1: pi-class glutathione-S-transferase; OGG1: 8-oxoguanine DNA glycosylase 1; ERCC6: excision repair 6 chromatin remodeling factor; LOXL1: lysyl oxidase-like 1; RAC1: rac family small GTPase 1; GSTM1/5: glutathione S-transferase isoform mu1/mu5; AMD: age-related macular degeneration; ECM: extracellular matrix; GCD2: granular corneal dystrophy type 2; 5hmC: 5-hydroxymethyl cytosine; NF-kB: nuclear factor kappa-light-chain-enhancer of activated B cells; PEX: pseudoexfoliation syndrome; RB: retinoblastoma; ROS: reactive oxygen species.
